# Deoxynivalenol and Nivalenol (2nd edition) [Assuring the Maximum Level of Deoxynivalenol in Wheat] (Natural Toxins and Mycotoxins)

**DOI:** 10.14252/foodsafetyfscj.D-20-00031

**Published:** 2020-12-25

**Authors:** Food Safety Commission of Japan

## Abstract

Food Safety Commission of Japan (FSCJ) was requested by the Ministry of Health, Labour and Welfare (MHLW) to conduct a risk assessment of deoxynivalenol (DON) to assure the maximal level for DON in foods. Previously, FSCJ had conducted a self-tasking risk assessment of DON and nivalenol (NIV) in 2010. In the current 2nd edition, only the assessment of DON has been revised. Grains contaminated with DON may be also contaminated with its derivatives, namely, 3-acetyldeoxynivalenol (3-Ac-DON), 15-acetyldeoxynivalenol (15-Ac-DON) and deoxynivalenol-3-glucoside (DON-3-glucoside). However, these substances orally ingested are rapidly biotransformed into DON. Therefore, FSCJ identified the total DON (sum of DON and its derivatives) to be assessed. The toxicity of DON was assessed based on the data of absorption-distribution-metabolism-excretion (ADME), acute toxicity, sub-acute toxicity, chronic toxicity, carcinogenicity, reproductive/developmental toxicity, genotoxicity, and immunotoxicity. DON was considered to have no significant genotoxic activity *in vivo*. The no-observed-adverse-effect level (NOAEL), based on the two-year chronic toxicity study in mice, was set at 0.1 mg DON/kg bw/day. By applying an uncertainty factor (UF) of 100, the TDI for DON was determined as 1 µg /kg bw/day. The average estimated exposure levels of total DON were 0.09 µg /kg bw/day and 0.22 µg/kg bw/day in the whole population and the 1-6 years group, respectively, by the Monte-Carlo method. The average exposure level in Japan was thus judged to be below the TDI, although a chance to exceed the TDI remains possible in the 1-6 years group depending on eating habits and DON contamination. For NIV, the genotoxic property was not able to be assessed due to the limited availability of the experimental data. No carcinogenic effect was observed in a two-year chronic toxicity study in mice, and the International Agency for Research on Cancer (IARC) also classifies *Fusarium* spp toxins including NIV to be in group 3. FSCJ thus judged that TDI can be set for NIV. Based on various toxicity studies, the TDI of NIV was determined at 0.4 µg/kg bw/day by taking into account of LOAEL 0.4 mg NIV/kg bw/day in a subacute toxicity study in rats with 90-day oral administration and UF of 1,000. The exposure level of NIV in Japan was estimated to be below the TDI. FSCJ judged it’s unlikely that NIV intake leads to adverse health effects in general population.

## Conclusion in Brief

Food Safety Commission of Japan (FSCJ) conducted a self-tasking risk assessment of deoxynivalenol (DON) and nivalenol (NIV) in 2010^1^^)^. In 2018, FSCJ was requested by the Ministry of Health, Labour and Welfare (MHLW) to conduct a risk assessment of DON based on article 24, paragraph (1), item (i) of the Food Safety Basic Act, for the purpose of assuring the maximal level for DON in foods. Accordingly, FSCJ prepared the 2nd edition of the risk assessment report to verify and organize new findings. In the 2nd edition, only the assessment of DON but not of NIV has been revised.

Grains contaminated with DON may also be contaminated with 3-acetyldeoxynivalenol (3-Ac-DON), 15-acetyldeoxynivalenol (15-Ac-DON) and deoxynivalenol-3-glucoside (DON-3-glucoside). These substances orally ingested are rapidly biotransformed into DON. These are thus assumed to be metabolized and excreted in ways similar as observed with DON. Therefore, FSCJ identified DON as the hazard to be assessed without considering toxicities of individual substances, the sum of DON and the derivatives (total DON) was thus used for the assessment.

The toxicity of DON was assessed based on scientific data of absorption-distribution-metabolism-excretion (ADME), acute toxicity, subacute toxicity, chronic toxicity, carcinogenicity, reproductive/developmental toxicity, genotoxicity, and immunotoxicity.

Major effects of DON observed in animal toxicity studies included emesis, decreased feed intake, suppressed body weight, and influence on immune system. At high doses, fetal toxicity and teratogenicity were detected. While confined levels of chromosomal abnormality were observed in several genotoxicity studies, DON showed no carcinogenicity on mice in a two-year chronic toxicity study. Based on these results, DON was considered to have no significant genotoxic activity *in vivo*. FSCJ, therefore, judged that it is appropriate to establish a tolerable daily intake (TDI) for DON.

The no-observed-adverse-effect level (NOAEL) for the two-year chronic toxicity study in mice was 0.1 mg DON/kg bw/day, based on suppression of body weights at the lowest-observed-adverse-effect level (LOAEL). FSCJ considered that this point of departure (POD) was appropriate to establish TDI among the various toxicity studies. By applying an uncertainty factor (UF) of 100, the TDI for DON was determined as 1 µg/kg bw per day^2^^)^.

The Monte-Carlo method was used to estimate exposure levels of DON and the derivatives in Japan, based on the intake data of wheat-containing food items. Wheat is reasonably considered as the main source of intakes of total DON. The average estimated exposure level of total DON in the whole population was 0.09 µg/kg bw/day, and the 95th percentile was 0.38 µg/kg bw/day. The corresponding exposure level in the 1-6 years group was 0.22 µg/kg bw/day, and the 95th and the 99th percentile values were 0.94 µg/kg bw/day and 1.86 µg/kg bw/day, respectively.

The average exposure level of total DON in Japan was judged to be below the TDI at present. Nonetheless, the exposure level in the 1-6 years group was close to the TDI and might have a chance to exceed the TDI depending on the eating habits and DON contamination. Extra exposure to DON in grain other than wheat might not be excluded.

FSCJ, therefore, considered that the risk management agencies should be aware of the reduction of the risk to a technically feasible level. In addition, the risk management agencies should endeavor to collect or investigate data which allow a precise estimation intake of total DON in order to eliminate uncertainties based on methodological characteristics and data collection.

Major effects of NIV observed in animal toxicity studies included decreased feed consumption, suppressed body-weight, and influence on the immune system. At high doses, embryotoxicity was detected. Chromosomal abnormality was reported in some genotoxicity studies. Genotoxic properties of NIV was not able to assess due to the limited availability of the experimental data. Since NIV showed no carcinogenic effects in a two-year chronic toxicity study in mice, and the The Insecticide Resistance Action Committee (IARC) has classified toxins produced by Fusarium spp. including NIV as Group 3, FSCJ judged that it allows to establish a TDI for NIV.

Among the various toxicity studies, the lowest value was observed at 0.4 mg NIV/kg bw/day as LOAEL (decreased WBC counts observed in a subacute toxicity study in rats with 90-day oral administration). By applying an UF of 1,000 (the factor of 10 was added for LOAEL value from the subacute toxicity study), the TDI for NIV was determined as 0.4 µg/kg bw/day.

The exposure level to NIV in Japan had been estimated to be below the TDI^1^^)^. Therefore, FSCJ judged that it is unlikely that dietary intake of NIV causes adverse health effects in the general population of Japan.

A group TDI for DON and NIV was considered difficult to be established at present, because of the limited number of studies on the combined effects of the two toxins and the inconsistency of their results, and also of the uncertainty of the mechanism of action of each toxin.

As a future task, exposure assessment should be done, using upcoming data of intakes of the targeted toxins from wheat and the wheat products, which would be obtained as food consumption and individual content of the toxins supplied from the risk management agencies. If it is accomplished, the risk management agencies should consider adopting measures to reduce the exposure as with the reference to Codex Standards.
